# Metaproteomic analysis of human gut microbiota: where are we heading?

**DOI:** 10.1186/s12929-017-0342-z

**Published:** 2017-06-12

**Authors:** Pey Yee Lee, Siok-Fong Chin, Hui-min Neoh, Rahman Jamal

**Affiliations:** UKM Medical Molecular Biology Institute (UMBI), Universiti Kebangsaan Malaysia, 56000 Cheras, Kuala Lumpur, Malaysia

**Keywords:** Metaproteomics, Human, Gut Microbiota, Multi-omics, Technologies

## Abstract

The human gut is home to complex microbial populations that change dynamically in response to various internal and external stimuli. The gut microbiota provides numerous functional benefits that are crucial for human health but in the setting of a disturbed equilibrium, the microbial community can cause deleterious outcomes such as diseases and cancers. Characterization of the functional activities of human gut microbiota is fundamental to understand their roles in human health and disease. Metaproteomics, which refers to the study of the entire protein collection of the microbial community in a given sample is an emerging area of research that provides informative details concerning functional aspects of the microbiota. In this mini review, we present a summary of the progress of metaproteomic analysis for studying the functional role of gut microbiota. This is followed by an overview of the experimental approaches focusing on fecal specimen for metaproteomics and is concluded by a discussion on the challenges and future directions of metaproteomic research.

## Background

The human gastrointestinal (GI) tract is colonized by a highly diverse population of microbial community collectively known as the gut microbiota that play vital roles in maintaining human health [1]. Although relatively stable, alterations in the microbial consortium may occur due to factors such as aging, genetic mutations, inflammation and dietary change [2, 3]. Accumulating evidence indicates that imbalances in the microbial community or dysbiosis have potential adverse effects on human health, whereby such alterations are implicated in the development of numerous diseases, including metabolic disorders, inflammatory diseases and cancers [4].

Given the importance of gut microbiota in human health and disease development, it has been the subject of extensive investigations in recent years. The completion of a large-scale initiative known as the Human Microbiome Project in 2012 has marked an important milestone in the characterization of human microbiome in healthy individuals, which led to the establishment of a reference microbial genome database [5]. MetaHIT (Metagenomics of the Human Intestinal Tract) is another collaborative effort that aims to provide a reference catalog of gut microbiome in association with obesity and inflammatory bowel disease (IBD) [6]. Over the years, much effort has been devoted to determine microbiome composition in various diseased patients using metagenomic analysis to identify potential links between gut microbiota and diseases [7].

To date, the growing number of metagenomic studies have provided valuable insights into the structure and diversity of the gut microbial community and their genetic composition. However, it is important to note that there are several limitations in these studies. Firstly, these studies only uncovered gene sequences that were present but do not provide any clues regarding their actual gene or protein expression levels. Besides, metagenomic analysis does not discriminate between microbiota that are active, dormant or dead, as all microbial cells will be sequenced. Consequently, the precise functions of the gut microbiota are still largely unknown. Hence, other complementary approaches are needed to elucidate the functional capacity of human gut microbiota.

Over the past decade, metaproteomics which is defined as the large-scale profiling of the whole protein complement expressed by a complex microbial ecosystem [8], has been applied to analyze human gut microbiota. In comparison to metagenomics, metaproteomics is capable to reveal functional traits relevant to the underlying physiological states, thereby providing detailed insights into the connection between microbial diversity, functions and the impact on host biology. In this mini review, we summarize the recent progress of metaproteomic study in the context of human gut microbiota. Further, we discuss experimental approaches for metaproteomics and conclude by providing an outlook on the challenges and future research direction for metaproteomics.

### Metaproteomics of human gut microbiota

In a pioneering metaproteomic study of human gut microbiota, an initial input into the establishment and functional role of gut microbiota during early growth was obtained from the analysis of fecal microbiota from two infants [9]. Undoubtedly, the study is limited in the depth of analysis due to the absence of a suitable database. Despite the relatively simple fecal protein profile, only Bifidobacterial transaldolases protein was successfully identified by *de novo* sequencing back then. A few years along the road, with the likes of more powerful analytical tools and metagenome data availability, Young et al. recently provided a more detailed fecal metaproteome profile of a preterm infant, shedding light on the functional clues and host-microbiota interactions during early development [10].

As for the more complex human adult, an initial comprehensive fecal metaproteome analysis was performed on a healthy monozygotic twin pair [11] and subsequently followed by a high-throughput temporal analysis of intestinal metaproteomes between three female adults [12]. Both studies identified a common core proteome that was mainly mapped to metabolic pathways and detected a distinctive but relatively stable metaproteome for each individual. Notably, both studies also demonstrated discrepancies between protein levels predicted from the metagenomes and phylogenetic data with their actual abundances, hence further emphasize the essentiality of metaproteomics in understanding the protein expression dynamics.

As noted from prior study, the composition of mucosa-associated microbiota varies from those residing inside the lumen [13]. This finding was further corroborated in an animal study where dissimilar metaproteome profiles of mucus, gut content and feces were reported [14]. Moreover, different intestinal sites have been found to affect host protein diversity more profoundly than microbial colonization states [15]. By applying a different sampling approach, Li et al. analyzed mucosal lavage samples from different intestinal locations to assess metaproteome of mucosa-associated microbiota [16]. Significant differences in the mucosal metaproteome were noted between the proximal and distal colon, implying distinct functionality within specific intestinal niches. This approach would be useful to investigate spatial distribution and activities at the mucosa-lumen interface, but might not be practically feasible as being invasive and costly.

Apart from cataloguing gut microbial metaproteome in healthy individuals, comparative analyses to characterize differential protein profiles under altered physiological conditions have been performed. Antibiotics are known to cause disturbance in the microbiota composition and functions, which in turn will have potential consequences on health and disease [17]. A comprehensive multi-omics study revealed drastic changes in the protein profiles of the gut-associated microbiota following β-lactam therapy [18], which reflect functional adaptation of gut microbiota in response to the drug. Clearly, more studies are needed to understand how different antibiotics can shape the gut microbiota and the resulting effect on the host.

There is increasing evidence linking gut microbiota to diseases such as Inflammatory Bowel Disease (IBD), including Crohn's Disease (CD) and ulcerative colitis (UC), but the exact role of the microbiota is still unclear [19]. Erickson et al. studied alterations of gut microbiota in CD patients and found differentially expressed proteins that could be linked to the disease [20]. In another metaproteomic study that focused on mucosal-luminal interface in IBD, changes in the bacterial phylotypes were reported to be associated with host immune response and inflammation [21]. These findings provide an insight into host-microbiota interactions that may be correlated to the disease etiology. The disease-associated features were further corroborated by the discovery of distinct protein modules associated with IBD in the mucosal metaproteome of IBD patients, which were verified in an independent cohort [22].

Besides, altered intestinal microbiota has been implicated in the development of obesity but the mechanistic link remains obscure [23]. Striking enrichment of gut microbiota proteins involved in cell motility and vitamin B_12_ synthesis was reported in an obese adolescent subject, whereas the lean adolescent showed more active vitamin B_6_ synthesis [24]. Nevertheless, the results are rather preliminary as only one subject from each group was analyzed. In a recent fecal metaproteome study involving a larger group of individuals, Kolmeder et al. reported that the phylum Bacteroidetes was biologically more active in the obese group [25]. The authors have identified a subset of bacterial and human proteins that could be used to classify the subjects into their corresponding groups and unveiled functional shifts that could be correlated to obesity.

Functional associations of microbial imbalances with liver cirrhosis have also been characterized using metaproteomics. Remarkably, unique metaproteome and functional pathways were reported for the patients, highlighting distinct functional characteristics of the gut microbiota that could be linked to liver cirrhosis [26]. The results provide new insights into the host-microbe relationships in liver cirrhosis that warrant further investigation. Metaproteomics also has been applied to study the correlation between dysbiosis with cystic fibrosis. Debyser et al. detected significant differences in the gut microbial diversity and protein profiles, along with a strong increase in host acute phase proteins in the patients, which reflect the ongoing inflammatory condition [27]. The study also reported a set of host and microbial proteins that might serve as candidate biomarkers for cystic fibrosis.

In the trending research area of microbiota and probiotic, no conclusive data is observed regarding probiotic intervention on the host and microbial functionalities. Probiotic consumption did not cause significant alteration on the overall fecal protein profiles nor the functional pathways despite a reduction of fecal host proteins and concomitant increase in bacterial proteins [28]. In spite of some evidence for therapeutic effect shown by several probiotic studies in diseases, there are conflicting findings reported on the implications of probiotic supplementation in the healthy population [29].

**Fig. 1:**
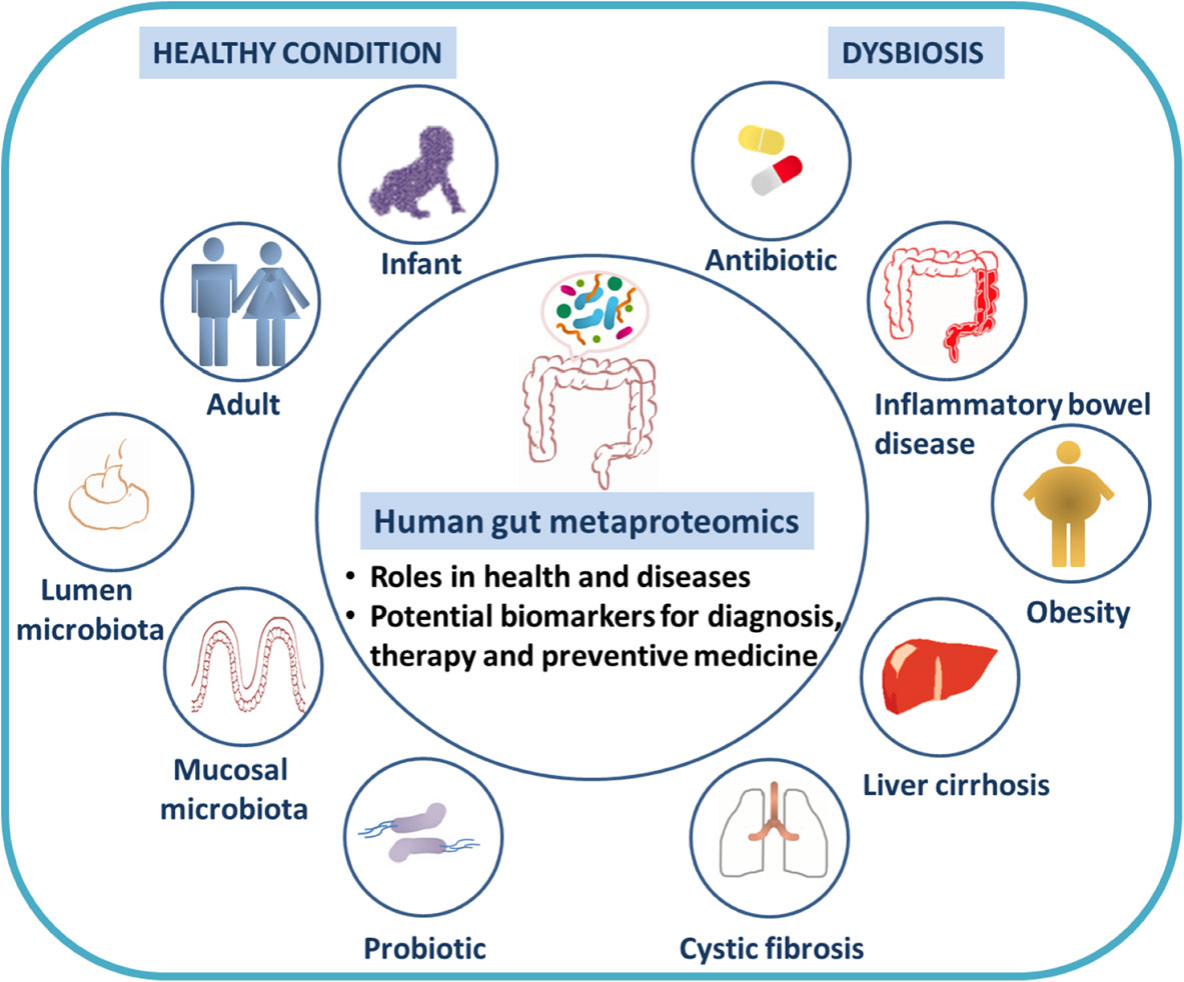
Applications of human gut metaproteomic study. Metaproteomic analysis has been employed to characterize functional roles of gut microbiota in healthy and disease conditions, which help to unravel the molecular mechanism underlying homeostasis and disease pathogenesis. Knowledge gained from metaproteomic study could be useful to devise strategies in disease prevention and management for improved human health

Overall, metaproteomic study is gradually gaining momentum to unravel the functionality of the complex microbial consortium. From understanding the role of microbiota in healthy individuals, the field has progressed to explore the functional profiles of dysbiosis in various diseases, as summarized in Table 1. Metaproteomics has the potential to dissect microbial functionality, which could help to understand the underlying pathophysiology and pave the way for targeted approach to improve health and disease (Fig. 1). Yet, we are only just beginning to decipher such associations and in addition to the diseases mentioned above, it would be interesting to explore other disease manifestations such as neurological disorders [30] and colorectal cancer [31].

**Table 1:** Summary of metaproteomic studies of human gut microbiota

Sample type	Subject	Separation method	Mass spectrometer	Database	No. of proteins identified	Reference
Feces	2 infants	2-D PAGE	MALDI-TOF	Public database	>200 spots, 1 protein identified	Klaassens et al., 2007
Feces	1 infant	2D-LC	LTQ Orbitrap	Databases from metagenomic sequences, microbial species, human and common contaminants	4,031 proteins	Young et al., 2015
Feces	1 monozygotic twin pair (adult)	2D-LC	LTQ Orbitrap	Databases from two human metagenomes, human database and common contaminants	446 human proteins 1,368 microbial proteins	Verberkmoes et al., 2009
Feces	3 healthy adults	1-DE and LC	LTQ Orbitrap	Databases from intestinal microbes, two metagenomes, human protein and food database	1,790 proteins	Kolmeder et al., 2012
Mucosal lavage	38 healthy adults	LC WCX magnetic beads	LTQ Orbitrap MALDI-TOF	Swiss-Prot	300 human proteins	Li et al., 2011
Feces	1 adult with antibiotic treatment	1-DE and LC	LTQ Orbitrap	Matched metagenome	3,011 proteins	Pérez-Cobas et al., 2013
Feces	6 monozygotic twin pairs (1 set healthy, 3 sets concordant twins with CD, 2 sets discordant twins)	2D-LC	LTQ Orbitrap	Matched metagenome and human microbial isolate reference genome database	2,904 proteins	Erickson et al., 2012
Mucosal lavage	First cohort: 3 healthy, 6 UC patients Second cohort: 13 healthy, 14 CD, 15 UC	WCX Protein Chip arrays	SELDI-TOF	Uniprot	589 proteins	Presley et al., 2012
Mucosal lavage	Discovery cohort: 51 (17 healthy, 13 UC, 21 CD) Validation cohort: 38 healthy	WCX magnetic beads LC	MALDI-TOF LTQ Orbitrap	Swiss-Prot	599 protein/peptide peaks	Li et al., 2016
Feces	1 lean and 1 obese teenager	1-DE and LC	LTQ Orbitrap	Matched and unmatched metagenomes	613 proteins	Ferrer et al., 2013
Feces	9 normal, 4 overweight, 16 obese	1-DE and LC	Orbitrap	In-house metaproteome database	893,007 MS/MS spectra	Kolmeder et al., 2015
Feces	3 liver cirrhosis patients and their corresponding spouse	1-DE and LC	Quadrupole-Orbitrap	UniProt	5,020 proteins	Wei et al., 2016
Feces	15 children with cystic fibrosis and their unaffected siblings	1-DE and LC	Ion trap-FTICR	NCBI	1,676 proteins	Debyser et al., 2016
Feces	16 healthy adults (8 probiotics, 8 placebo)	1-DE and LC	LTQ Orbitrap	In-house metaproteome database	4,966 peptides	Kolmeder et al., 2016
Feces	1 healthy adult	LC	LTQ Orbitrap	UniProtKB Swiss-Prot	3,911 proteins (not centrifuged) 4,587 proteins (centrifuged)	Tanca et al., 2015
Feces	2 premature infants	2D-LC	LTQ Orbitrap	In-house metaproteome database	807 and 342 proteins (without filtering) 1264 and 1012 proteins (with double filtering)	Xiong et al., 2015b
Feces	2 healthy adults	1-DE and LC	LTQ Orbitrap	Matched metagenomes Synthetic metagenomes (iterative search)	2,331 and 1,870 peptides 5,010 and 3,542 peptides	Rooijers et al., 2011
Feces	4 healthy volunteers	LC	LTQ Orbitrap	Metagenome databases UniProt-based databases All bacterial sequences	>10,000 peptides	Tanca et al., 2016
Feces Mucosal lavage	8 mice 8 pediatric volunteers	LC	Q Exactive	Microbial gene catalog databases Target-decoy database	30,749 mouse protein groups 19,011 human protein groups	Zhang et al., 2016b
Mucosal lavage	5 teenagers	LC	LTQ Orbitrap	In-house Human Intestinal Microbial Protein Database Target-decoy database	4,014 protein groups quantified	Zhang et al., 2016a

*1-DE* one-dimensional electrophoresis, *2-D PAGE* two-dimensional polyacrylamide gel electrophoresis, *2-D LC* two-dimensional liquid chromatography, *FTICR* Fourier transform ion cyclotron resonance, *LC* liquid chromatography, *LTQ* linear trap quadrupole, *MALDI-TOF* matrix-assisted laser desorption/ionization time-of-flight, *NCBI* National Center for Biotechnology Information, *SELDI-TOF* surface-enhanced laser desorption/ionization time-of-flight, *WCX* weak cation exchange

### Experimental considerations for metaproteomics

Metaproteomics workflow typically includes sample collection, protein extraction, fractionation, mass spectrometry (MS) analysis and database searches [32]. For human gut microbiota study, fecal and mucosal lavage samples are commonly employed to characterize global proteome of the entire gut and the mucosa interface, respectively. This mini review will focus on fecal sample as it is more widely used for metaproteomics. Sample storage is a crucial yet sometimes overlooked step in metaproteomics. Several independent studies have revealed that different storage temperatures may introduce considerable alterations to the microbial profiles and highlighted that proper storage is critical to maintain sample stability [33–35]. Moreover, it was found that frozen intact fecal material was more stable than frozen extracted proteins, hence is recommended for long-term storage [36].

Apart from storage, sample processing is another key step in metaproteomics. Sample preparation protocol primarily depends on the research questions, which isolate either host or microbial proteins or both. Most previous studies have focused on proteins of microbial origin and employed centrifugation to remove other interfering substances. However, it was observed that despite greater microbial protein identifications, the centrifugation step caused considerable protein loss due to non-specific removal of microbial cells, which led to bias in the analysis [37]. Conversely, stool without pretreatment provides a better representation of the microbial proteins and allows concurrent analysis of human proteins. This highlights the importance of careful consideration in selecting a suitable approach for sample processing. Alternatively, a double filtering separation step has been shown useful to deplete human proteins for selective enrichment of microbial proteins, which was demonstrated to enhance proteome coverage by facilitating the identification of low-abundance proteins [38].

Next, efficient protein extraction from the complex microbial samples is critical to allow accurate representation of the intracellular protein content. In the metaproteomic analysis of environmental samples, different protein extraction methods have been shown to isolate different subset of proteins with only minimal overlap, which underlines the importance of selecting appropriate protocol to obtain optimal protein sample [39]. For gut microbiota study, several studies have indicated that mechanical disruption by bead beating was an efficient protein extraction method, particularly for lysing Gram-positive bacteria [40, 41]. Thus far, there is a major gap in the characterization of extracellular proteins that may serve as major mediators of host-microbiota interactions. The challenge to capture the secreted proteins from a complex ecosystem is huge, as consideration for intracellular protein removal either from the host or microbiota must be taken into account. Fecal samples may provide sufficient protein yield for this kind of secretome study but protein loss is inevitable given the necessity of an extensive clean-up procedure that follows due to the nature of the sample itself. Fecal proteins may also undergo some alteration along the intestine. Lichtman et al. described the enrichment of secreted gut luminal proteins from feces that can be applied to facilitate analysis of secreted host proteome [42]. Other than that, targeted analysis of specific subcellular fraction such as membrane proteins and post-translational modifications are also likely to provide additional functional insights.

To date, MS remains as the analytical platform of choice for metaproteomics. Prior to MS analysis, extensive fractionation using multidimensional LC separations (GeLC-MS/MS or 2D-LC-MS/MS) is particularly useful to reduce sample complexity and improve protein identification. The final and fairly demanding stage for metaproteomics is data analysis. Several software tools such as Pipasic [43], MetaProteomeAnalyzer [44] and Unipept [45] have been developed to facilitate metaproteomic data analysis. One of the key elements for a successful metaproteomic study is the availability of a relevant database for mass spectra searching. Strategy using either matched or unmatched metagenomes has been successfully employed for metaproteomic protein identifications [24, 46]. Furthermore, iterative workflow using synthetic metagenome generated from known gut microbiota has been shown successful to enhance protein identifications [47].

The choice of database is a critical factor in data analysis. Parallel use of multiple databases to improve protein yields may be the way forward as demonstrated by Tanca et al. in which the use of different databases in gut microbial metaproteome data analysis has led to complementary identification of unique peptides [46]. More recently, a data analysis pipeline coupling publicly accessible gene catalog databases with iterative database searching known as MetaPro-IQ was introduced by Zhang et al. [48]. The pipeline enabled efficient identification and quantification of over 120,000 peptides corresponding to >30,000 protein groups from human and mouse gut microbial metaproteome. To date, it represents the most extensive metaproteome coverage and appears to be a promising approach for future metaproteomic study.

### Challenges and future directions

Despite the great potential of metaproteomics to decipher the diverse roles of microbial members within the human host, there are many obstacles that need to be surmounted. First and foremost, inherent sample complexity associated with the highly diverse microbial community is among the major hurdles for metaproteomic study. The vast protein dynamic range often hinders the detection of low-abundant proteins from the minor species. This problem could be partly alleviated by applying different fractionation and enrichment strategies, such as capillary and microchip methods [49] to reduce sample complexity and increase protein detection, but at the expense of increased cost and analysis time.

Beyond protein identification, quantitative analysis is important to determine key microbial players that contribute to metabolic functions [50]. However, given the enormous species and metabolic diversity, robust approaches for quantitative metaproteomic are still lacking. Protein-based stable isotope probing (protein-SIP) has been reported as a powerful technique in environmental studies to decipher metabolic activity of distinct microbial members [51]. Concerning gut microbiota, label-free quantification is the most common strategy adopted in the aforementioned metaproteomic studies, but has limited accuracy. The most recent take on quantitative metaproteomics of human gut microbiota is the application of metabolic labeling for improved peptide quantification [52]. This newly introduced method hold the potential to facilitate future metaproteomic study.

There are also technical limitations implicated in metaproteomic analysis. Standardized procedures for metaproteomics are yet to be established, which might lead to suboptimal findings and hinder inter-laboratory comparison. Moreover, existing analytical platforms are also limited in terms of their sensitivity to analyze protein sample with such a wide dynamic range. With the emerging technology advancement in mass spectrometry such as the data-independent acquisition strategy (MS^E^ and SWATH), reproducibility of protein quantification and the depth of proteome analysis were significantly improved [53]. Apart from that, identification of proteins from the complex microbial consortium, which may harbor up to hundreds or thousands of species, has also proven to be a difficult task. Lack of complete genome sequences for the highly heterogeneous microbial community, particularly the poorly characterized and uncultivable species poses a big challenge for researchers. Nonetheless, the availability of sequence data from the blooming metagenomic studies and new analysis softwares are expected to counteract these issues.

Additionally, it is evident that sample complexity and inter-individual variation in gut microbiota are extensive [16]. It is also important to note that host and microbiota interactions involve delicate interplay between factors such as age, genetics, immunity and dietary habits, thus these could act as potential confounding variables. Clearly, studies involving a larger set of well-defined subjects are neccesary to capture a more accurate functional input of the gut microbial ecosystem. Rodent and gnotobiotic animals can be custom-designed to circumvent obstacles related to human study, thus represent valuable models for studying gut microbiota and have been employed in several metaproteomic studies [14, 42, 54].

Despite all of the limitations, metaproteomic research has already led to some remarkable discoveries on the functional features of gut microbiota. With the ongoing development to address those challenges, we envision that the field will further advance in the future. Furthermore, integration of metaproteomic with other omics approaches is expected to provide a more comprehensive and meaningful elucidation of the microbial ecosystem.

## Conclusion

With the growing interest to understand the link between gut microbiota in health and diseases, metaproteomic analysis is instrumental to characterize the activity and functional pathways of the microbial community. Although challenging, further advances in sample preparation methods, development of more sophisticated analytical tools alongside with the availability of relevant software and databases are expected to facilitate the progress of metaproteomics in the coming years. By harnessing the power of the emerging technologies, it is anticipated that more details on the microbial functionality and their connection with human host will be uncovered in the future.

## Abbreviations

CD: Crohn's Disease
GI: Gastrointestinal
IBD: Inflammatory Bowel Disease
MetaHIT: Metagenomics of the Human Intestinal Tract
MS: Mass spectrometry
protein-SIP: Protein-based stable isotope probing
UC: Ulcerative colitis
